# Evidence in Support of the Independent Channel Model Describing the Sensorimotor Control of Human Stance Using a Humanoid Robot

**DOI:** 10.3389/fncom.2018.00013

**Published:** 2018-03-20

**Authors:** Jantsje H. Pasma, Lorenz Assländer, Joost van Kordelaar, Digna de Kam, Thomas Mergner, Alfred C. Schouten

**Affiliations:** ^1^Department of Biomechanical Engineering, Delft University of Technology, Delft, Netherlands; ^2^Department of Neurology, University Clinics Freiburg, Freiburg, Germany; ^3^Sensorimotor Performance Lab, University of Konstanz, Konstanz, Germany; ^4^Department of Biomechanical Engineering, Institute for Biomedical Technology and Technical Medicine (MIRA), University of Twente, Enschede, Netherlands; ^5^Department of Rehabilitation, Donders Centre for Neuroscience, Radboud University Medical Center, Nijmegen, Netherlands

**Keywords:** balance control model, system identification, parameter estimation, robotics, human balance control

## Abstract

The Independent Channel (IC) model is a commonly used linear balance control model in the frequency domain to analyze human balance control using system identification and parameter estimation. The IC model is a rudimentary and noise-free description of balance behavior in the frequency domain, where a stable model representation is not guaranteed. In this study, we conducted firstly time-domain simulations with added noise, and secondly robot experiments by implementing the IC model in a real-world robot (PostuRob II) to test the validity and stability of the model in the time domain and for real world situations. Balance behavior of seven healthy participants was measured during upright stance by applying pseudorandom continuous support surface rotations. System identification and parameter estimation were used to describe the balance behavior with the IC model in the frequency domain. The IC model with the estimated parameters from human experiments was implemented in Simulink for computer simulations including noise in the time domain and robot experiments using the humanoid robot PostuRob II. Again, system identification and parameter estimation were used to describe the simulated balance behavior. Time series, Frequency Response Functions, and estimated parameters from human experiments, computer simulations, and robot experiments were compared with each other. The computer simulations showed similar balance behavior and estimated control parameters compared to the human experiments, in the time and frequency domain. Also, the IC model was able to control the humanoid robot by keeping it upright, but showed small differences compared to the human experiments in the time and frequency domain, especially at high frequencies. We conclude that the IC model, a descriptive model in the frequency domain, can imitate human balance behavior also in the time domain, both in computer simulations with added noise and real world situations with a humanoid robot. This provides further evidence that the IC model is a valid description of human balance control.

## Introduction

Human balance control helps us to keep our body in an upright position during daily life activities. In human balance control several systems are involved, like the sensory systems, the nervous system, and the muscles, which interact continuously with each other (Horak, 1997). Visual, proprioceptive (muscle spindles and Golgi tendon organs) and vestibular cues are integrated by the nervous system to obtain body orientation with respect to the visual scene, the support surface and gravito-inertial space, respectively. The nervous system integrates these sensory cues to generate a desired torque signal realized by the muscles. This torque results in a corrective movement to bring the body toward the desired upright position. The body position and velocity thus changes and the new body position and velocity are again sensed by the sensory systems. Thus, balance control can be described as a closed loop control system (Collins and De Luca, 1993; Peterka, 2002; van der Kooij et al., 2005).

To describe and understand the interaction between the underlying systems, human balance control models are useful (Engelhart et al., 2014). The Independent Channel (IC) model is a frequently used linear parametric model describing the interaction between the underlying systems during stance in a closed loop (Peterka, 2002). In this model the human body is modeled as a single inverted pendulum and each sensory system is modeled as a separate feedback channel with a weighting factor, which reflects the contribution of each sensory system during stance. Peterka (2002) quantified the changes in sensory contributions depending on the balancing situation, showing that changes in sensory contributions, referred to as sensory reweighting, plays an important role in human balance control.

The IC model is a simple descriptive model of the balance behavior and is formulated by a transfer function in the frequency domain, which allows easy and fast implementation of parameter estimation (Schoukens et al., 2004). To describe dynamic balance behavior, a non-parametric approach in the frequency domain can be used in combination with a continuous periodic perturbation with specific frequency content for system identification (Johansson and Magnusson, 1991; van der Kooij et al., 2005). Fitting the frequency domain model on the measured balance behavior then provides a limited set of physiologically interpretable parameters describing the underlying systems (Peterka, 2002; Kiemel et al., 2011). Theoretically, however, frequency domain models are a global description over the whole frequency range and may show small imperfections at specific frequencies. They may even include unstable subsystems in the fitting procedure. Furthermore, the IC model is used on averaged data and therefore in approximately noise free situations. As noise is inherent in human balance control, the IC model may not be able to stabilize this noisy system. In addition, the IC model may miss some essential details, as it is a simplified representation of human balance control and the human body is modeled as a linearized inverted pendulum (Peterka, 2003). Thus, the IC model may not always be a valid representation of the human balance behavior in real world situations.

In this study, we evaluated the validity of the IC model (i.e., a frequency domain model) by testing the model in time domain simulations with added noise and in a real world environment with the humanoid robot PostuRob II (Hettich et al., 2014) to show the functionality of a frequency domain model in the time domain and in real world situations. The robot has human-like anthropometrics, pneumatic muscle actuation and noisy and inaccurate sensors. The human IC model was used to control the robot, i.e., to generate a torque command based on the weighted sensory information. Comparing the robot's balance behavior to human balance behavior in similar experimental conditions is an important validation of the model. The real world environment may provide additional insight into human balance control and the robustness of the model.

## Materials and methods

### Independent channel model

**Figure 1:**
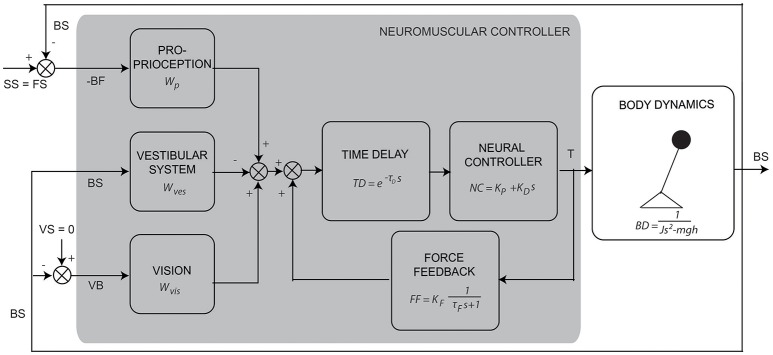
Schematic representation of the human balance control (adapted from Peterka, 2003). The human body is represented by an inverted pendulum (body dynamics, BD) controlled by the neuromuscular controller producing a torque (T). The neuromuscular controller consists of the neural controller (NC), represented by a PD controller, a lumped time delay (TD) and the sensory feedback “channels” with their weighting factors [i.e., for proprioception (*W*_*p*_), vision (*W*_*vis*_), and vestibular system (*W*_*ves*_)] and force feedback (FF). The external perturbation is a support surface (SS) rotation around the ankle joint axis. It changes the orientation of the feet in space relative to the horizontal (FS) and therefore changes the body angle with respect to the feet (BF, sensed by proprioception). This results in a conflict between the proprioceptive information and the information sensed by the vestibular system [i.e., body angle in space relative to the gravitational vertical (BS)] and vision [i.e., the visual surround orientation relative to the body (VB)] and evokes a change of the body angle in space (BS).

The IC model (see Figure 1) describes the aforementioned process of balance control in the form of a simplified descriptive linear model. The model consists of a single inverted pendulum (body dynamics: BD) controlled by a feedback mechanism with a PD controller (neural controller: NC) and a time delay (TD). The sensory integration mechanism consists of a weighted sum of the sensory contributions, where the weights always sum to unity (Peterka, 2002). The first contribution is the relative orientation of the body to the feet (BF), sensed by the proprioceptive system (*W*_*p*_). The proprioceptive signal BF refers here to an abstract internal representation of body orientation with respect to the feet instead of the ankle joint angle itself (Peterka, 2002; Mergner, 2010). The second is the body orientation with respect to the space vertical (BS), sensed by the vestibular system (*W*_*ves*_). The third is the visual surround orientation relative to the body (VB), sensed by the visual system (*W*_*vis*_). The weighted signals are summed with a low-pass filtered positive force feedback (FF; Peterka, 2003), which accounts for a relatively good compensation of body lean at low frequencies. Together they provide an error signal as feedback into the PD controller (see Figure 1). In the time delay (TD) all delays in the loop are lumped, including muscle activation, neural delays, and processing time.

### Study design

**Figure 2:**
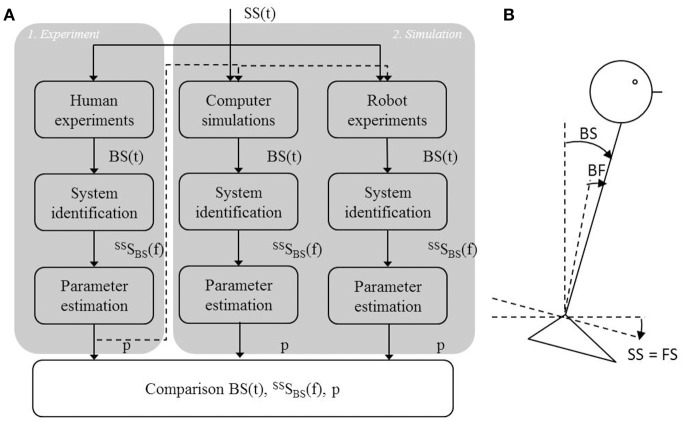
Flowchart of the study set up and the experimental set up. **(A)** First, a human experiment (1) was performed using support surface rotations [*SS*(*t*)] as shown by the experimental set up **(B)**, in which the SS is rotated around the ankle joint axis. This rotation changes the feet orientation in space (FS) relative to the earth horizontal. Based on the body sway responses, *BS*(*t*), the Frequency Response Function [FRF, ^SS^S_BS_(f)] was calculated, which allowed to estimate the control parameters (*p*) that described the balance behavior. These parameters were then implemented into the models used for the computer simulations and the robot experiments (2) using the same perturbation signal as in the human experiment. *BS*(*t*) obtained from the simulations and robot experiments was again used for system identification and parameter estimation and for describing the balance behavior in terms of FRFs and estimated parameters. Finally, balance behavior obtained from computer simulations and robot experiments were compared with balance behavior obtained from the human experiments, using the time series, FRFs, and estimated parameters.

To validate the IC model, three steps were performed (Figure 2A). First, human balance behavior of healthy participants was obtained in human experiments using support surface rotations in space (SS) and body-in-space sway (BS) measurements (Figure 2B). SS rotations evoke body sway through changes of the body angle relative to the feet (BF) sensed by proprioception and the resulting sensory conflict with the body angle in space (BS) sensed by the vestibular system and by vision in a stationary visual surround (Figure 2B). The human balance behavior was analyzed in the frequency domain using system identification techniques. Parameter estimation was performed using the IC model (Peterka, 2002) resulting in parameters describing the underlying human balance control system.

Secondly, the time domain computer simulations using the IC model with added noise and the estimated parameters from human experiments were performed. Thirdly, the model was implemented in the humanoid robot to test the IC model under real world conditions. To compare the human balance behavior in the time domain, the same perturbations as in the human experiments were used. Again, system identification and parameter estimation were used to describe the balance behavior and to estimate the model parameters representing the balance behavior obtained from the computer simulations and robot experiments. Finally, the balance behavior obtained from the computer simulations and robot experiments were compared with the balance behavior obtained in the human experiments.

### Human experiments

#### Participants

Seven healthy young participants (5 males, 2 females, age 26.1 ± 2.1 years, height: 1.79 ± 0.09 m, mass: 77.7 ± 10.8 kg) were included in the study. The participants gave written informed consent prior to participation. The protocol was approved by the medical ethics committee of the Medical Spectrum Twente, Enschede, the Netherlands and was in accordance with the Declaration of Helsinki.

#### Apparatus

A Bilateral Ankle Perturbator (BAP) (Forcelink B.V., Culemborg, the Netherlands) was used to apply support surface (SS) rotations around the ankle joint axis (Schouten et al., 2011). The actual angles of rotation of the SS on the BAP were measured.

The body kinematics of the lower and upper body were measured in anterior-posterior direction using two draw-wire potentiometers (Sentech SP2, Celesco, Chatsworth, CA, United States) by connecting them to the participant's trunk and hip. Together with the SS rotation, the body kinematics were measured using a Matlab interface with a sample frequency of 1,000 Hz.

#### Perturbation signal

**Figure 3:**
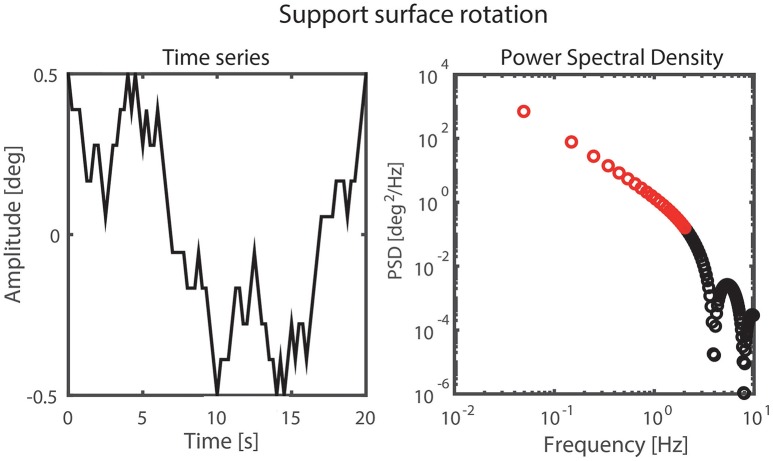
Perturbation signal and the corresponding power spectral density on the odd harmonics with the analyzed frequencies shown in red.

A pseudorandom ternary sequence (PRTS) with 80 states and a time increment of 0.25 s was generated, resulting in a signal with a period of 20 s (Davies, 1970; Peterka, 2002). This signal was used as SS angular velocity of both the left and right SS simultaneously. Integration of this signal provided the perturbation signal of the SS rotation with a wide spectral bandwidth where only the odd harmonics contain signal power (Peterka, 2002; Figure 3). The even harmonics were not excited by the perturbation and were used to detect nonlinearities in the output (Pintelon and Schoukens, 2001). Each trial consisted of six complete repetitions of the perturbation signal resulting in a trial duration of 2 min. The signal was applied with peak-to-peak amplitudes of 0.5 and 1 degrees.

#### Procedure

During all experiments the participants stood on the BAP wearing socks. The participants were instructed to stand with their arms crossed at chest level and to keep both feet on the support surface. The perturbations were applied at both amplitudes during eyes open and eyes closed conditions, resulting in four trials of 2 min each. Before recording, the participants were given sufficient time to familiarize with the perturbation (~10 s). The participants wore a safety harness to prevent falling, which did not constrain normal body sway and did not provide support or body orientation information.

#### Preprocessing

Data analysis was performed with Matlab (The Mathworks, Natick, MA, United States). Leg and hip angles were calculated using the potentiometer data and the attachment height of the potentiometers, resulting in the segment angle of the legs relative to the vertical and the joint angle of the trunk relative to the legs. The body-in-space sway (BS), taken as the angular displacement of the whole body Center of Mass without feet (CoM) relative to the vertical, was calculated using the leg and hip angles and body anthropometrics obtained from Winter et al. (1990). The time series of the body sway and the actual SS rotation were used for further analysis.

#### System identification and parameter estimation

The time series of the body sway and SS rotation were segmented into six data blocks of 20 s (i.e., the length of the perturbation signal) and were transformed to the frequency domain. The periodic part of the frequency coefficients was calculated by averaging the frequency coefficients across the six data blocks. The Cross Spectral Density (CSD) of the body sway and the perturbation and the Power Spectral Density (PSD) of the perturbation were calculated.

To test for nonlinearities in the body sway as response to the perturbation, the PSD of the body sway on the odd and even harmonics were calculated. Effect of nonlinearities within each tested condition was quantified by the percentage of total body sway power on the even harmonics, where the perturbation had no power. Thus, a higher percentage indicates excitation of body sway at frequencies which were not excited by the perturbation, and therefore more non-linearities. A low percentage of nonlinearities is a prerequisite for the application of linear system identification techniques.

Next, the Frequency Response Functions (FRFs) representing the sensitivity function of the SS rotation to the body sway were estimated by dividing the CSD of the body sway and the perturbation by the PSD of the perturbation (Equation 1; Peterka, 2002; van der Kooij et al., 2005). Only the excited frequencies (i.e., odd harmonics) were analyzed. In case the amount of nonlinearities is low, the response on the excited frequencies (i.e., odd harmonics) represents the total balance behavior. The function is given by

(1) $$\begin{array}{l} H_{\exp}\left(f\right)=\Phi_{SS,BS}\left(f\right)\cdot {\left[\Phi_{SS,SS}\left(f\right)\right]}^{-1} \end{array}$$

where Φ_*SS, BS*_ and Φ_*SS, SS*_ represent the CSD and PSD, respectively. The magnitude and phase represent the relation between the perturbation and body sway per frequency in terms of amplitude ratio and timing, respectively.

The coherence reflects the amount of body sway evoked by the perturbation on the excited frequencies, i.e., the linear response, and decreases with noise and nonlinearities (Pintelon and Schoukens, 2001). The coherence is given by

(2) $$\begin{array}{l} \gamma_{SS,BS}^{2}\left(f\right)={\left|\Phi_{SS,BS}\left(f\right)\right|}^{2}{\left[\Phi_{SS,SS}\left(f\right)\Phi_{BS,BS}\left(f\right)\right]}^{-1} \end{array}$$

where Φ_*BS, BS*_ represents the PSD of the body sway. The coherence varies between 0 and 1, with a coherence close to one reflecting a good signal to noise ratio and linear behavior.

The FRFs and coherences were obtained with nonparametric analysis and were averaged across the participants for each condition resulting in four FRFs and coherences (i.e., for the 0.5 and 1 degrees perturbation amplitudes with eyes open and eyes closed), which were used for further validation.

The IC model was fitted on the estimated balance behavior during each condition, represented by the FRFs averaged across participants, using the theoretical transfer function of the IC model, as presented in Equation (3), to obtain parameters describing the balance behavior (Peterka, 2003). To characterize the postural effects evoked by the SS rotation around the ankle axis, the proprioceptive weight (*W*_*p*_) was estimated from

(3) $$\begin{array}{l} H_{est}\left(f,p\right)=\frac{BS\left(f\right)}{SS\left(f\right)}=\frac{W_{p}\cdot NC\cdot TD\cdot BD}{1-FF\cdot NC\cdot TD+NC\cdot TD\cdot BD} \end{array}$$

where *NC* represents the neural controller, *TD* the time delay, *BD* the body dynamics and *FF* the force feedback. *f* represents the frequency vector and *p* the model parameters, namely the mass (*m*), CoM height (*h*), moment of inertia (*J*), proprioceptive weight (*W*_*p*_), the reflexive stiffness (*K*_*P*_), the reflexive damping (*K*_*D*_), time delay (τ_*D*_), force feedback time constant (τ_*F*_) and force feedback gain (*K*_*F*_) (Figure 1). As the sum of the weights equals one, in case of eyes closed, *W*_*vis*_ is zero and *W*_*ves*_ can be calculated by 1 – *W*_*prop*_. With the eyes open, the sum of *W*_*vis*_ and *W*_*ves*_ equals 1 – *W*_*prop*_ (see Peterka, 2002), where visual and vestibular weight cannot be separated mathematically.

The body mass, CoM height and moment of inertia were used as fixed parameters. The CoM height and moment of inertia were calculated using the method of Winter et al. (1990). The model was fitted on the FRFs (0.05–2.05 Hz) of the averaged human experimental data using a nonlinear least-square fit (Matlab function: lsqnonlin) by minimizing the sum squared error (E), equation (5), in which more more weight was given to the low frequencies and the frequencies with higher coherence (Equation 4).

(4) $$\begin{array}{l} \varepsilon \left(f,p\right)=\sqrt{\frac{\gamma_{SS,BS}^{2}\left(f\right)}{1+f}}\cdot |\log\left(\frac{H_{\exp}\left(f\right)}{H_{est}\left(f,p\right)}\right)| \end{array}$$

(5) $$\begin{array}{l} E=\frac{1}{N}\varepsilon {\left(f,p\right)}^{T}\varepsilon \left(f,p\right) \end{array}$$

$\gamma_{SS,BS}^{2}$ represents the averaged coherence between the SS rotation and body sway, *H*_*exp*_ the averaged experimental or simulated sensitivity function, *H*_*est*_ the estimated sensitivity function based on the estimated model parameters (*p*) and *N* the number of frequencies.

The quality of the model fit was represented by the Variance Accounted For (*VAF*) (Equation 6) identifying how well the model describes the observed time series averaged across data blocks and participants. The *VAF* is given in percentage; 100% indicates that the model accounts fully for the experimental data. A lower *VAF* indicates deviations between the model and the time series averaged across data blocks and participants.

(6) $$\begin{array}{l} VAF=1-\frac{\overset{T}{\underset{t=0.01}{\sum }}|BS_{\exp,t}-BS_{est,t}{|}^{2}}{\overset{T}{\underset{t=0.01}{\sum }}|BS_{est,t}{|}^{2}}*100\% \end{array}$$

where *BS*_exp, t_ represents the body sway measured in the experiment and *BS*_*est, t*_ represents the body sway obtained from simulations with the estimated model parameters.

The Standard Error of the Mean (SEM) of each parameter represents the sensitivity of the error (ε, Equation 4) to changes in parameters and was calculated using the diagonal of the estimated covariance matrix (*P*) obtained during the parameter estimation procedure (Ljung, 1999; Equation 7).

(7) $$\begin{array}{l} \overset{\wedge }{P}=E{\left(J^{T}J\right)}^{-1} \end{array}$$

in which *J* is the Jacobian (matrix of partial derivatives of each parameter's prediction error ε given in Equation 4) and *E* the sum squared error. Since the parameters are estimated using the averaged experimental FRFs, the SEM reflects the precision of the estimated parameters and not the amount of variation of the model parameters.

### Computer simulations

The IC model as described in section Independent Channel Model and Figure 1 was implemented in Simulink, Matlab (The Mathworks, Natick, MA, United States) with added pink noise to mimic sensory and motor noise (van der Kooij and Peterka, 2011). The human body was modeled as a single inverted pendulum and all parameters (Equation 3, Figure 1) were set to the values found in the human experiments. The same perturbation signal (section Perturbation Signal) and analyses (section System Identification and Parameter Estimation) as used in the human experiments were applied resulting in time series, FRFs, and estimated parameters describing the balance behavior simulated by the computer.

### Robot experiments

#### PostuRob II

To imitate human balance control in a real world situation, the humanoid robot PostuRob II was used (Hettich et al., 2014). The robot was constructed with human-like anthropometric parameters (mass: 51 kg, CoM height above feet: 0.97 m, moment of inertia: 40 kgm^2^) consisting of trunk, leg, and feet segments interconnected with two actuated ankle and hip joints (hip joints were fixed during this study).

The sensory signals of the vestibular system, joint torque, joint angular position, and velocity were measured using mechatronic sensors. The technical analog for the vestibular system are accelerometers and gyrometers, where the signals are processed to provide body angular velocity and angle with respect to the gravitational vertical, and linear acceleration in the sagittal plane (Mergner et al., 2009). In the current study only the angular orientation with respect to gravity was used in the robot experiments as the IC model only uses this signal.

Torque commands were sent to the robot to actuate artificial pneumatic “muscles” at the ankle joints (Type MAS20, FESTO AG & Co.KG, Esslingen, Germany). An inner torque control loop ensured that the actual torque matches the torque commands. A real time PC with Simulink (Real-Time Windows Target, The Mathworks Inc., Natick, USA) was used as the control module, running the compiled IC model.

#### Apparatus

A custom-built motion platform (Hexapod, Stuart principle; Mergner et al., 2003) was used to apply SS rotations around the ankle axis. The same perturbation signals as in the human experiments were used (see section Perturbation Signal). The body kinematics of the lower and upper body of the robot were measured in anterior-posterior direction using an optical motion capture system with two active markers attached to the robot's hip and shoulder, respectively (Optotrak 3020; Waterloo, Canada). The body kinematics together with the actual angle of rotation were measured using custom made software written in LabView (National Instruments, Austin, USA) with a sample frequency of 100 Hz and were stored for further analysis.

#### Procedure

The same procedure was performed with the PostuRob II as in the human experiments; the experiments consisted of four 2-min long trials with SS rotations and different perturbation amplitudes. Before each trial the parameters estimated from the human experimental data for each condition were implemented in the robot. This step was necessary, as the IC model requires a different set of model parameters for each condition. The neural controller parameters were corrected to account for the difference in mass and CoM height between human participants and the robot. Note that the robot had no visual sensor. Instead, the artificial vestibular sensor was also used for the robot experiments to mimic eyes open conditions, where only the model parameters were adjusted according to the changes identified in the human participants. Data were analyzed according to the procedures described in sections Preprocessing and System Identification and Parameter Estimation.

## Results

### Time series

**Figure 4:**
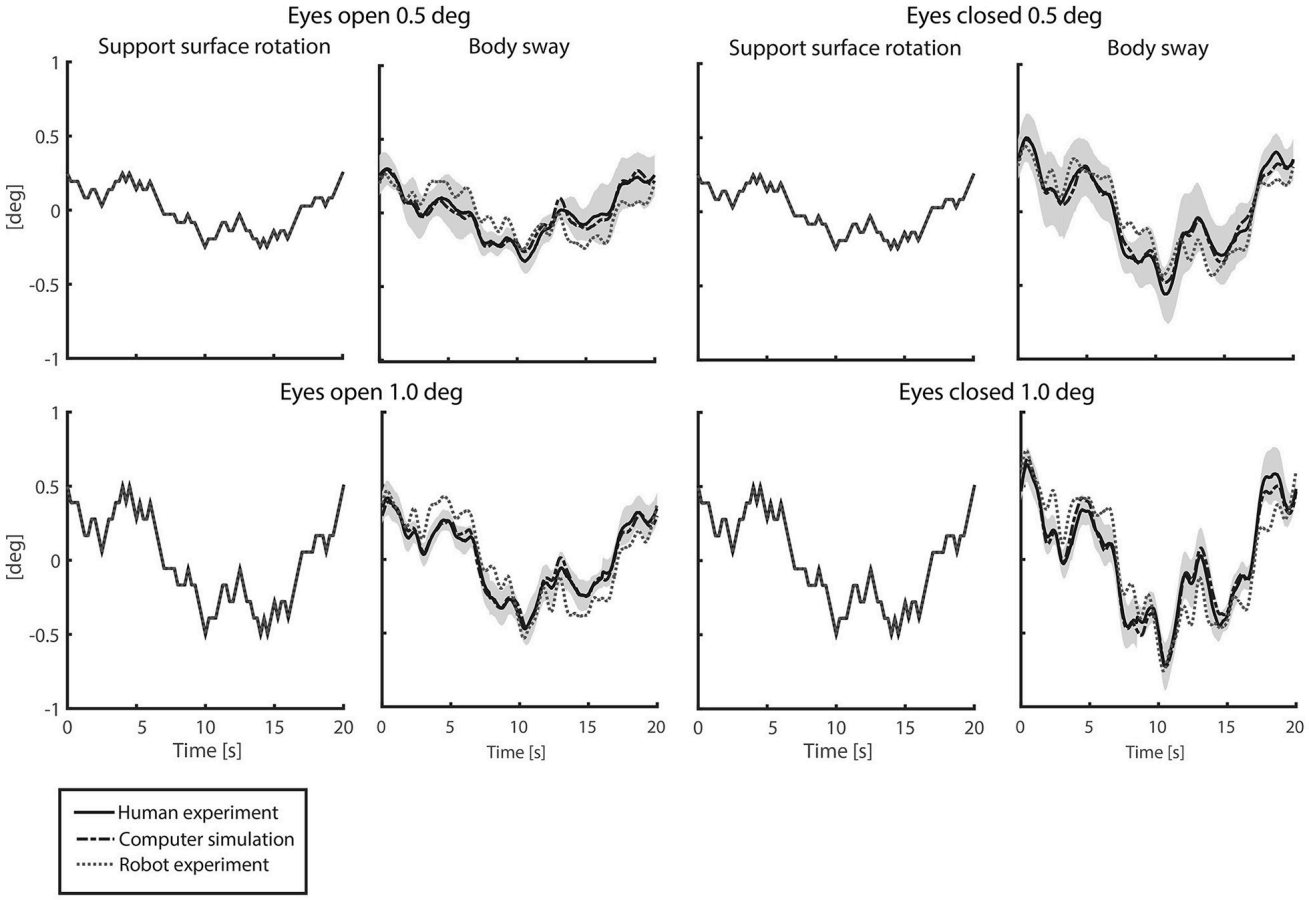
Time series of support surface rotation and body sway for the four conditions investigated in the human experiments, computer simulations, and robot experiments. The four conditions refer to the two amplitudes (0.5 and 1 degrees peak-to-peak perturbation amplitude) and the eyes open (EO; left) and eyes closed (EC; right) condition. The data are shown for the human experiments, averaged across participants (solid black lines) presented by the mean value with standard deviation (shaded), the computer simulations (dash-dotted gray lines), and the robot experiments (dotted gray lines).

Figure 4 shows the time series of the averaged body sway for the human experiments, computer simulations, and robot experiments for each condition. Sway responses of computer simulations and robot experiments followed the general pattern of the human sway responses. The *VAF*s between the human experiments and the computer simulations (“*VAF*_*S*_”) were in the range of 94.1–98.7% for all conditions and in the range of 62.9–79.0% for the robot experiments (“*VAF*_*R*_”; Table 1). For both the computer simulations and the robot experiments, the *VAF* tended to increase with increasing perturbation amplitude and was higher in eyes closed, as compared to eyes open conditions. Computer simulations and robot experiments were robust with respect to the noise and the inaccuracies, and control stability was maintained throughout all conditions.

**Table 1:** Differences between human experiments and computer simulations or robot experiments per experimental condition given for the time series and estimated parameters.

	**Computer simulation vs. human experiment**				**Robot experiment vs. human experiment**			
	**0.5**	**1**	**0.5**	**1**	**0.5**	**1**	**0.5**	**1**
	**EO**	**EO**	**EC**	**EC**	**EO**	**EO**	**EC**	**EC**
**Time series**								
Variance Accounted For (*VAF*, %)	94.2	97.2	94.1	98.7	63.2	62.9	69.2	79.0
**Estimated parameters**								
Relative mean difference (%)	16.9	14.6	13.3	10.7	27.3	26.3	19.8	13.6

*Data are presented for the four conditions with perturbation amplitude of 0.5 and 1 degrees peak-to-peak and eyes open (EO) versus eyes closed (EC)*.

### Frequency response functions

**Figure 5:**
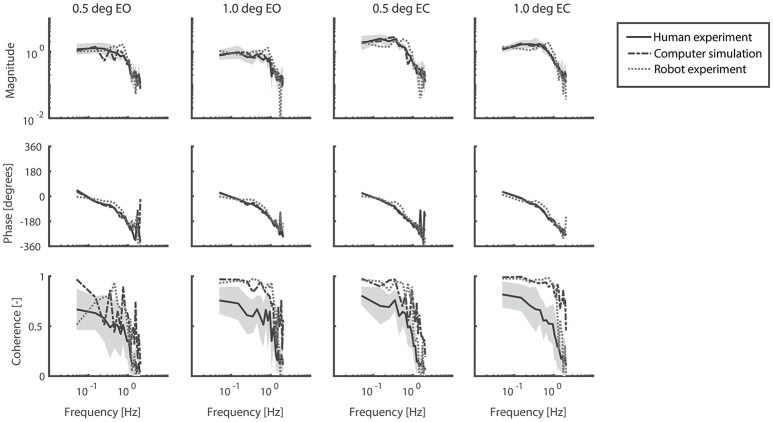
Frequency Response Functions representing the sensitivity functions of the support surface rotation to body sway. The four conditions refer to the two amplitudes (0.5 and 1 degrees peak-to-peak perturbation amplitude) and the eyes open (EO) and eyes closed (EC) condition. Shown are the magnitude, phase and coherence for the human experiments (averaged across participants, solid black lines) presented by mean values with standard deviations (shaded) and correspondingly for computer simulations (dash-dotted gray lines) and robot experiments (dotted gray lines).

Figure 5 shows the FRFs with the corresponding coherence for each condition of the human experiments, together with those obtained from the computer simulations and the robot experiments. In general, the pattern of the FRFs and the changes across conditions were similar in humans, computer simulations, and robot experiments. Simulations and the robot experiments showed some differences in the magnitude as compared to humans with the largest difference between robot and human experiments at about 0.7 Hz. The coherence was considerably larger in simulations and the robot as compared to humans, indicating differences in the noise properties. The percentage power of the body sway on the even harmonics varied between 1.8 and 7.4% of total body sway power in the human experiments and therefore did not show strong nonlinearities.

### Estimated parameters

The quality of the model fits was represented in the time domain by the *VAF*. For the human experimental data the *VAF* varied between 98.0 and 99.1%, for the simulated data between 91.9 and 99.0% and for the robot experiments between 93.4 and 98.3%, indicating only small deviations between the measured (or simulated) data and the fitted model.

**Figure 6:**
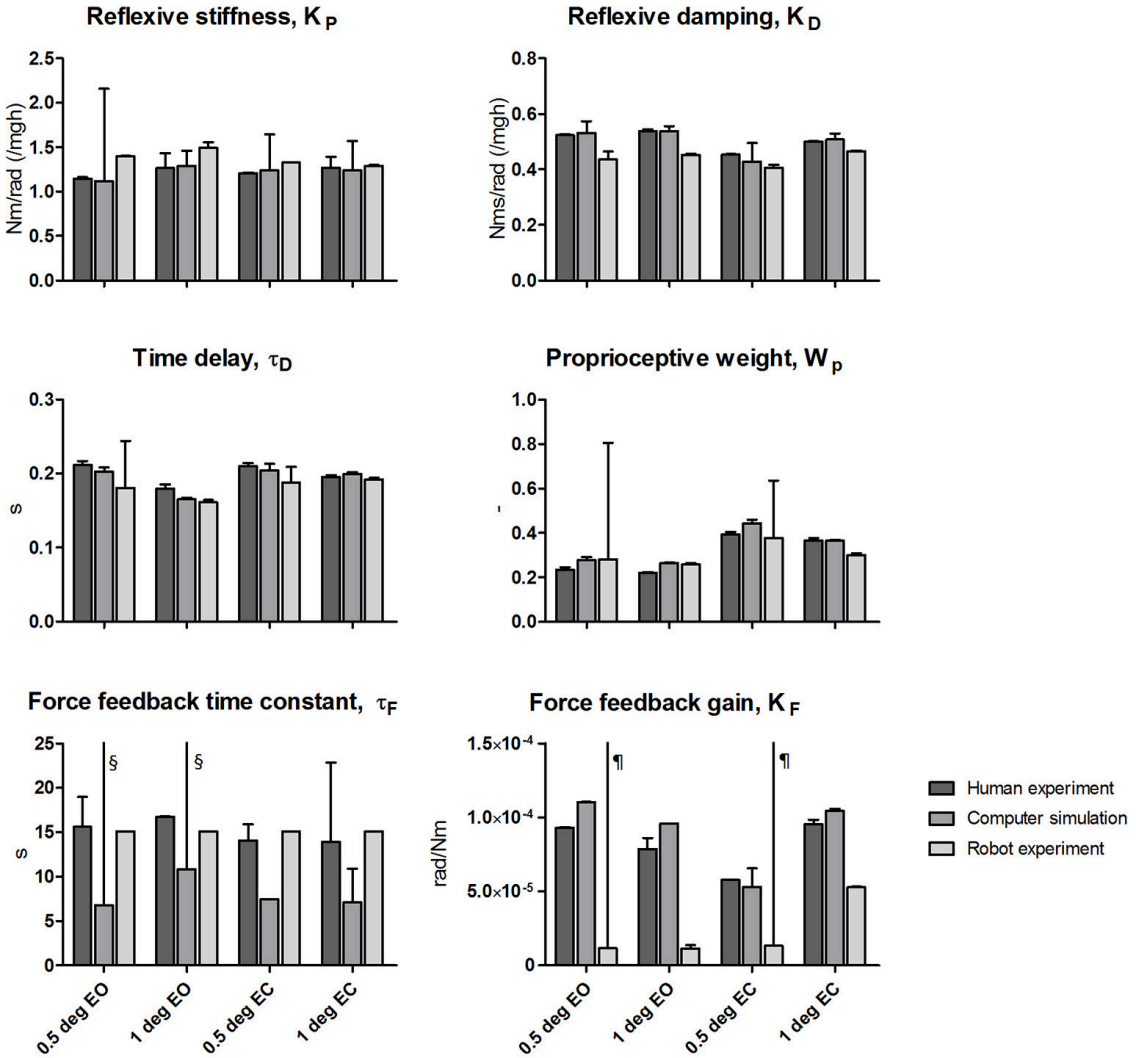
Estimated parameters of the Independent Channel model of each condition for the human experiments, computer simulations and robot experiments. The estimated values are shown together with the Standard Error of the Mean (SEM) representing the reliability of the estimates obtained from fitting. Results of the four tested conditions [0.5 and 1 degrees peak-to-peak perturbation amplitude with eyes open (EO) and eyes closed (EC)] are shown. The reflexive stiffness and damping were normalized to the gravitational stiffness (i.e., Center of Mass height multiplied by mass and the gravitational constant g). ^§^SEM values > 190 s; ^¶^SEM values > 5.0 × 10^−4^ rad/Nm.

Figure 6 shows the estimated parameters with the corresponding SEM of the human experiments, the computer simulations and the robot experiments. The estimated SEM values indicating the sensitivity of the fitting error to changes in the given parameter were low for most parameters. Exceptional large SEM values stand out in the simulations for the reflexive stiffness (only eyes closed 0.5 degrees) and the force feedback time constants, and in the robot experiments for the time delays, the proprioceptive weight, and the force feedback gain values obtained for the small perturbation amplitudes (0.5 degrees).

The mean relative differences between the parameters is presented in Table 1. The differences between the parameters of the computer simulations and the parameters of the human experiments were in the range of 10.7–16.9%. The differences between the parameters of the robot experiments and the human experiments were in the range of 13.6–27.3%. Again, the differences decrease with increasing perturbation amplitude and with closing the eyes.

Overall, the estimated parameters of the human experiments, the computer simulations, and robot experiments are comparable for all conditions. Clear differences were found in the force feedback time constant and gain, which also show a large SEM. Furthermore, the reflexive stiffness, and reflexive damping showed a larger difference between the human experiments and the robot experiments compared to the other parameters.

## Discussion

In this study we validated the IC model, a commonly used descriptive model in the frequency domain, in the time domain using computer simulations with added noise and in the real world using robot experiments. The results show that both the computer simulations and robot experiments can reproduce human balance behavior, where computer simulations described the human sway responses better compared to the robot. The model simulations showed that the IC model is stable in the time domain with added noise, which adds an important aspect to the descriptive nature of this frequency domain model. Furthermore, the robot, controlled by the IC model, maintained the desired upright position, which showed that the IC model is robust enough to deal with the real-world properties of the robot (i.e., human-like anthropometrics, noisy and inaccurate sensors, and mechanical dead zones).

### Experimental balance behavior

The experimental balance behavior was estimated using a linear approach. The low percentage power of the body sway of the healthy participants at even harmonic frequencies shows that no considerable nonlinearities that are effective across frequencies were found within the steady state of one condition. The absence of such nonlinearities allowed the use of a linear approach by only analyzing the excited frequencies (i.e., odd harmonics).

A linear model was fitted for each condition of the experimental data to describe the balance behavior. The high *VAF* of the model fits indicate that the model explains the data well. The estimated parameters obtained from human experiments are comparable with previous studies and show sensory reweighting, i.e., a change in the use of sensory information (e.g., decrease in proprioceptive weight) with changing perturbation amplitude and sensory condition (i.e., with increasing perturbation amplitude and opening the eyes; Peterka, 2002; Cenciarini and Peterka, 2006; Pasma et al., 2015). Also an increase in reflexive stiffness was found with increasing perturbation amplitude, which is in agreement with previous studies (Peterka, 2002).

### Replication of human balance behavior with computer simulations

The results revealed small differences between the time series of the human experiments and those of the computer simulations. Small differences between time series, FRFs, and estimated parameters were expected, as pink noise was implemented in the computer simulations. Furthermore, we observed that the IC model described the human responses in the high frequency range not as accurately as in the low and mid frequency ranges. This can be explained by the used prediction error function, which gives more weight to the frequencies with a high coherence.

These results provide evidence that the IC model, a frequency domain model, is able and therefore valid to be used to control a system in the time domain. The stable computer simulations showed that the frequency domain model does not represent an unstable subsystem and is able to tolerate physiologically plausible noise without loss of balance.

Also, the estimated parameters of the human experiments and the computer simulations are comparable. An exception is the force feedback time constant, which mainly affects balance behavior at low frequencies. The large SEM value indicates that the estimate is less reliable. Due to the length of the perturbation signal (i.e., 20 s), the perturbation contained little information in the low frequency range, resulting in the observed low reliability in the estimates. Notably, however, this parameter had only small influences on the time series and FRFs.

### Replication of human balance behavior with robot experiments

The main purpose of the study was to show the functionality of the IC model in real world situations using robot experiments. Similar approaches have been used to test other balance control concepts, like the Disturbance Estimation and Compensation concept or the Eigen movement concept (Hettich et al., 2014; Alexandrov et al., 2017). Here, the IC model was able to control the robot in the time domain when adjusting the estimated neural controller parameters (i.e., the reflexive stiffness and damping) to the mass and weight of the robot. The robot's control was stable across conditions and in the presence of manually applied pushes (results not shown). In response to the pushes, the robot showed a compliant behavior (relatively small resistance to the push), which is related to the low loop gain used in the IC model and an important characteristic of healthy human balancing.

The differences between the robot experiments and the human experiments in the time series, the FRFs, and the estimated parameters were larger than those between computer simulations and human experiments. One likely reason is that the mechanical components of the robot introduce additional inaccuracies due to dead zones, friction, etc., which remained unconsidered in the robot's control model. The ability of the IC model to stabilize the robot despite these unconsidered effects suggests a considerable robustness of the control mechanism. This robustness is also a major aspect in human balance control, which lends further support to the evidence that human balance control can be explained by such a simple feedback mechanism as described by the IC model.

A difference between the robot experiments and the human experiments concerned a peak around 0.7 Hz in the magnitude of the FRFs. The peak decreased with increasing perturbation amplitude and increasing torque level. Manual changes in the model parameters and additional experiments (not shown in the results) suggest that this peak might be due to the activation dynamics of the robot's actuators in terms of a resonance peak. As the peak decreased with increasing perturbation amplitude, this suggests a nonlinear behavior of the robot's actuation.

The peak around 0.7 Hz also may explain the higher reflexive stiffness and lower reflexive damping estimates for the robot experiment data as compared to the human experiment data. Furthermore, a clear difference was found for the force feedback gain. As already mentioned above, the force feedback estimates showed a high SEM value and primarily affects the low frequency range, where the differences between the FRFs and between the estimations of the force feedback parameters were largest.

### Decrease in differences with increasing perturbation amplitude

The results show that, overall, the differences between the human experiments and the computer simulations on the one hand and the human experiments and the robot experiments on the other hand decreased with increasing perturbation amplitude, as shown by the *VAF*s and the relative mean difference of the parameters shown in Table 1. In the computer simulations, these differences can be attributed mainly to the noise injected into the model. The amplitude of the noise was kept constant across the conditions. This means that with a higher perturbation amplitude the noise had less influence on the time series (resulting in a better signal to noise ratio) and therefore had less influence on the FRFs and the estimated parameters. This may explain why the differences between the human experiments and the computer simulations became less with increasing perturbation amplitude and why the SEM of the estimated parameters became smaller.

The argument could also hold for the robot experiments if one assumes that with increasing perturbation amplitude, the effects of the sensory and motor noise became relatively smaller, and similarly also the effects of the activation dynamics and mechanical inaccuracies. The reduced difference with increasing perturbation amplitude and with closing the eyes suggests that the robot controlled by the IC model is able to reproduce human balance behavior.

### Limitations

The IC model is a simplification of the human balance control, in which the human body is modeled as an inverted pendulum pivoting around the ankle joint axis and the equations of motion are linearized. The model can be used to describe balance behavior in the frequency domain at a specific operating point as long as the balance conditions are not changed and the deviations from this point are small. Therefore, the model can only be used during steady state conditions, e.g., within one amplitude, which might also be possible with other models.

In case of larger perturbations, which result in larger deviations and also in rotation around the hip joints in addition to the rotation around the ankle joints, the IC model would miss essential details as suggested by studies which used balance control models that incorporate also the hip joints, modeling the human body as a double inverted pendulum (Qu and Nussbaum, 2012; Boonstra et al., 2013; Hettich et al., 2014; Engelhart et al., 2015; Hwang et al., 2016). These models are able to identify both the control of the upper and lower body separately and the intersegmental coupling.

The somewhat lower coherence of the human experimental data likely originates from noise and variability present in the measured time series given the low amount of nonlinearities in the system. The coherence is plausible since the sway amplitude evoked by the small perturbation was comparable to the sway amplitude not evoked by the perturbation (i.e., spontaneous sway in quiet stance). The coherence values are also comparable to other studies (Pasma et al., 2012, 2015; Boonstra et al., 2013). Despite the somewhat lower coherence in the human experimental data, the sensitivity function described the linear balance behavior in the humans rather well and can be explained well by the IC model, as shown by a high variance accounted for.

Intrinsic dynamics of the passive tissue and tendon structures were neglected in the formulation of the IC model. This simplification was implemented based on earlier studies suggesting that the intrinsic dynamics contribute only about 10% to the overall torque generated by the active muscle contractions (Peterka, 2002; Maurer et al., 2005; Cenciarini and Peterka, 2006; Assländer et al., 2015; Vlutters et al., 2015; Wiesmeier et al., 2015). Furthermore, previous studies showed that it is difficult to experimentally determine the intrinsic dynamics during balance control (Peterka, 2002; Pasma et al., 2015; Engelhart et al., 2016). As our model was able to explain the obtained sensitivity functions well without the intrinsic dynamics, we decided to dismiss them here.

## Conclusions

This study showed that the IC model, a descriptive linear model in the frequency domain, is able to imitate human balance behavior in both the time and frequency domain, this both in computer simulations and robot experiments. Therefore, the IC model represents a good descriptor of human balance control. The capability to tolerate noise and keep the robot in an upright position, while being externally perturbed, indicates that the IC model is robust in the time domain and in a real world situation.

The IC model may help in the future to obtain further insights into human balance control and to develop better and more human-like balance control mechanisms for robotic assistive devices such as exoskeletons. Furthermore, the robot implementation is useful for educational purposes, as it opens the possibility to experience the functionality of the IC model in a direct interaction with the human-like behaving robot. It remains to be shown to what extent the IC model can help to detect and classify changes underlying impaired balance control.

## Author contributions

DdK and JvK: performed the human experiment and collected the data; JP and LA: performed the robot experiments; JP: performed the computer simulations and analyzed the data. All authors contributed to the interpretation of the data. JP and LA: made a draft of the manuscript; DdK, JvK, TM, and AS: critically revised the manuscript. All authors approved the final version of the manuscript for submission.

### Conflict of interest statement

The authors declare that the research was conducted in the absence of any commercial or financial relationships that could be construed as a potential conflict of interest. The handling editor is currently editing a Research Topic with one of the authors AS, and confirms the absence of any other collaboration.
